# Security and Violence Perception of Medical Interns during Social Service Practice in Mexico

**DOI:** 10.3390/ijerph19010318

**Published:** 2021-12-29

**Authors:** Margarita L. Martinez-Fierro, Miguel A. Ramirez-Madrigal, Rosa Martha Covarrubias-Carrillo, Lorena Avila-Carrasco, Virginia Flores-Morales, Oscar G. Meza-Zavala, María de León-Sigg, Sodel Vázquez-Reyes, Alejandro Mauricio-González, Perla Velasco-Elizondo, Idalia Garza-Veloz

**Affiliations:** 1 Academic Unit of Human Medicine and Health Sciences, Autonomous University of Zacatecas, Zacatecas 98040, Mexico; m.angelmadrigal22@gmail.com (M.A.R.-M.); rosmarth.covarru@gmail.com (R.M.C.-C.); doctoralac@gmail.com (L.A.-C.); virginia.flores@uaz.edu.mx (V.F.-M.); medicinahumana.uaz@gmail.com (O.G.M.-Z.); vazquezs@uaz.edu.mx (S.V.-R.); amgdark@uaz.edu.mx (A.M.-G.); pvelasco@uaz.edu.mx (P.V.-E.); 2 Academic Unit of Electrical Engineering, Autonomous University of Zacatecas, Zacatecas 98040, Mexico; mleonsigg@uaz.edu.mx

**Keywords:** social service medical intern, Zacatecas, violence, assaults, medicine

## Abstract

The increase of insecurity levels in Mexico, as well as the fact that violence is a frequent experience among health personnel, motivated this study whose purpose was to evaluate the perception of security and violence that social service medical interns (SSMI) had on the institutions and localities where they carried out their social work and make visible the main types of violence to which they were exposed. This was a cross-sectional study, based on a perception survey self-administered to 157 SSMI from Zacatecas, in Mexico. A high proportion of the participants (75.8%) stated that they were victims of violence, describing 134 incidents; however, only 33.6% of SSMI made an official report. The reported incidents were related to organized crime (31.9%), verbal violence (20.6%), violence by the authorities (14.7%) and sexual harassment (11.8%). One hundred percent of the victims of sexual harassment were women (*p* = 0.039). According to the above, it is a priority to generate strategies to prevent and reduce the risk of exposure to the violence generated in the medical units and communities where SSMI carry out their activities as medical graduates, as well as, to efficiently process formal violence reports to promote a safe environment that favors the fulfillment of the practice of SSMIs in Mexico.

## 1. Introduction

*Social Service* in Mexico is a period of public service that graduate students are required to provide, generally immediately after graduation, to apply their knowledge to serve the public good. In Mexico, the practice of social service for medical interns started in 1936. The first social service program ran in the Faculty of Medicine of the National Autonomous University of Mexico (UNAM). This, in agreement with what was stated in a reform of articles 3 and 5 of the Political Constitution of the United Mexican States and 24 of the General Education Law, that medical interns had to complete a one-year period of unpaid social service [1,2,3,4,5]. Today, about 16,500 medical interns participate each year in a process, where a set of available positions to perform social service in medical units are offered. This process occurs in each university in Mexico where positions are offered considering the transcript grades of medical interns [6,7]. Unfortunately, violence is a common phenomenon among health services personnel [8,9]. Achieving the goals of social service practice highly depends on doing it in an environment where physical and mental health, as well as safety and violence prevention of SSMIs and people working in medical units, is promoted [8].

The World Health Organization (WHO) defines violence as the intentional use of physical force or power, threatened or actual, against oneself, another person, or against a group or community, that either result in or has a high likelihood of resulting in injury, death, psychological harm, maldevelopment or deprivation (limitation to perform any activity or decision making with freedom). Worldwide, acts of violence are the cause of about 1.6 million deaths [10]. Several types of violence have been described and are classified according to the nature of the act, like physical, sexual, or psychological, including deprivation or neglect (left alone or not supported in their daily activities) [11,12]. This classification considers the implications of violence for public health and its focus is primarily on domestic and gender violence, which are common in Mexico. However, violence exerted by organized crime has been gaining strength as one of the most important factors that impact security conditions in Mexico [13]. In addition to the above, in an environment of insecurity, the perception of abusive situations in medical students during their undergraduate education is a controversial topic, as it exposes the multiple difficulties of dealing with violence between medical students, which are different at different stages of their studies and can change later on in a clinical environment [14,15]. Previous research on the quantitative evaluation of the perception of abuse of medical students shows that they encompass a very wide spectrum of abuse episodes that include physical abuse, sexual discrimination, as well as personal and professional undervaluation. All these episodes provoke in medical students a lack of sense of belonging in clinical settings [16]. In addition, studies have shown that 47% of SSMIs dropouts occur in medical units located in the states of Mexico with the highest rates of violence [17]. Risk factors that contribute to the likelihood of an SSMI becoming a victim of violence include the general and sanitary conditions of medical units (e.g., availability of air conditioning, toilets, drinking water, fences and dining rooms), safety conditions of medical units (e.g., equipment and tools, security services), risks of the physical environment, as well as SSMIs excessive workloads and hard schedules [18]. It is common that in unprotected rural areas of the north of Mexico, organized crime has routes of constant transit [19,20]; people living in these areas have a greater risk of suffering from violence. According to the National Institute of Statistics and Geography (INEGI), in 2018 Zacatecas state ranked 32nd in university student deaths from homicides. However, in 2019 the state ranked 14th, and in 2020 it ranked 10th [21]. As can be seen, university students are one of the most affected groups by the lack of safety, which represents another risk factor for SSMIs due to the kind of service they provide. Thus, in this study, we focus on measuring the perception of security and violence of medical interns during social service practice in medical units in locations of the state of Zacatecas, as well as identifying the factors that lead to such a perception. 

## 2. Materials and Methods

### 2.1. Study Design and Participants

The work we present in this paper is an observational, descriptive, and cross-sectional study. The population of the study included 157 SSMIs of the 2020–2021 class promotion, with at least 6 months of doing social service work. The inclusion criteria of the study were: SSMIs graduated from the Autonomous University of Zacatecas *“Francisco García Salinas”* or SSMIs graduated from the Autonomous University of Durango, Campus Zacatecas, who at the time of the study were doing their social service work in the state of Zacatecas and were part of either January 2020 to December 2021 or August 2020 to July 2021 promotion. Participants who explicitly asked that their responses be used for informational purposes, but not for research ones, were not considered in this study.

### 2.2. Description of the Intervention and Data Collection

An online survey, created with Google Forms, was used to collect data from SSMIs. The survey included 57 information items related to insecurity aspects of medical units and their geographic location; length and conditions of transport routes to medical units; work schedules, critical care protocols, working equipment, communication means and degree of marginalization. Besides, SSMIs were asked to rate on a scale from 1 to 10 the levels of security and insecurity of the medical units and their locations, where 1–2 = Safe, 3–4 = Moderately safe, 5–6 = Not very safe, 7–8 = Insecure and 9–10 = Highly insecure. Similarly, participants were asked to rate their perception of safety during their stay as SSMIs in medical units with Likert-type responses ranging from 1 = Not at all safe, 2 = Little Safe, 3 = Somewhat Safe and 4 = Safe. SSMIs completed the survey anonymously by themselves.

The norms NOM-005, NOM-009 and the guide titled “*Guía del Médico Pasante en el Servicio Social*” state the applicable specifications for social service work and positions [1,6,7,22]. Social service positions are typified into A, B and C, corresponding to the levels of marginalization–low, medium and high, respectively, of geographic locations of medical units [6,7]. Therefore, the number of hours and the role to fulfill of a social service medical intern (SSMI) will depend on the type of social service position assigned to each of them [6,7]. For example, social service positions of type A were defined as those in which SSMIs work from 6 to 8 h a day, from Monday to Friday, with the support of a principal doctor, this classification also applies to medical units with 24-h emergency care. In social service, positions of type A work include doctors who are federal workers, doctors with special health conditions and doctors who belong to institutional research lines [6,7]. Social service positions of type B were defined as those where SSMIs work 8 h a day, from Monday to Friday, in a medical unit with 3 or more cores and with the support of a principal doctor in the morning shift. Type C positions were specified as those where SSMIs cover 8 h a day and one 24 h shift of emergencies, in a medical unit with one to two cores, in a high marginalization location, with the SSMIs in charge of the medical unit, but always under supervision [23].

### 2.3. Data Analysis

The information obtained in this study was stored in a Microsoft 365 Excel spreadsheet. Data description was done by calculating mean, standard deviation, and percentage on various data. For graph generation, we used data frequencies and percentages. Chi-square was used to determine differences among proportions and odds ratio was used to compare the significant ones. Sigma Plot 12.0 v1.02 software was used to perform data analysis. Values of *p* < 0.05 were considered significant.

### 2.4. Ethical Considerations

The study presented in this paper is a cross-sectional one; then as it is stated in the General Health Law this study is considered “No risk” because no intervention or modification was made in the physiological, psychological, and social variables of the interviewed individuals. Their participation was voluntary and prior to their inclusion, they were asked to express written consent. The written consent was obtained as part of the electronic instrument that we used in this study. The confidentiality of personal data was guaranteed, and this study attended all recommendations stated in the Declaration of Helsinki, as well as the Regulations established by the Research Committee of the Academic Unit of Human Medicine and Health Sciences of the Autonomous University of Zacatecas (ID number: CI-R-00010-2021).

## 3. Results

A total of 157 SSMIs were included in this study; 86 (54.8%) of them were women. The average age of the respondents was 25 ± 1.18 years. One hundred forty-eight of the participants completed their undergraduate studies at the Autonomous University of Zacatecas and nine of them at the Autonomous University of Durango, Campus Zacatecas. The SSMIs included worked in one of the 157 medical units considered in this study. Forty-seven-point one percent of these medical units belonged to the Ministry of Health of Zacatecas (SSZ), 44.6% units to the Mexican Institute of Social Security (IMSS), 3.2% to the Institute of Social Security and Services for State Workers (ISSSTE) and 5.1% were specific medical areas at the Autonomous University of Zacatecas.

### 3.1. Description and Evaluation of Social Service Positions by Type

The social service positions occupied by the respondents were distributed as follows: 22 (14%) of type A, 47 (29.9%) of type B and 88 (56.1%) of type C. For these social service positions, the respondents noticed the existence of different conditions to those stated in the norms NOM-005, NOM-009 and the guide “*Guía del Médico Pasante en el Servicio Social*” [1,6,22]. For example, for type A social service positions, the respondents expressed that, 2.1% had different time schedules and responsibilities; for type B social service positions, 17% had different conditions and working hours than those stated by law; for type C social service positions, 9.1% had better conditions and corresponded to medical units located in places with lower levels of marginalization than those officially mandated.

### 3.2. Characteristics of the Infrastructure in Medical Units

Concerning the conditions of the physical infrastructure and the existence of security personnel in the medical units where social service work was performed, 105 (66.9%) had boundary fences or walls, 94 (59.9%) had protected windows, 22 (14.0%) had proper door locks, 16 (10.2%) had community security services, 3 (1.91%) had neighborhood security services and 2 (1.27%) had security personnel. Regarding the availability of communication means, 102 (64.96%) medical units had a computer with Internet access, 123 (78%) had access to a mobile phone, 61 (38.9%) had an on-site telephone line, 42 (26.8%) had a radio and 8 (5.1%) had no communication means.

### 3.3. Security Perception When Traveling from SSMIs’ Place of Living to Medical Units 

Regarding the perception of security when traveling from SSMIs’ place of living to medical units, 11 (7.0%) of the respondents considered their journey as Not at all safe, 58 (36.9%) as Not very safe, 61 (38.9%) as Somewhat safe and 27 (17.2%) as Safe. The causes related to poor security included, for the 4.5% of the respondents, the existence of organized crime checkpoints, the presence of corpses, barricades and/or organized crime persecutions at some point of their travel to medical units. The average duration of the travel from SSMIs’ place of living to medical units was 1 h and 39 min (range 90–120 min).

### 3.4. Knowledge of the Definition of Violence

Of the total respondents of SSMI who participated in the study, 93.0% had a general knowledge of the definition of violence, 3.8% said they did not know the definition of violence and 3.2% had a wrong understanding of the definition. Fifty-five-point four percent of the participants had an incomplete understanding of the definition of violence as they did not recognize omission, psychological manipulation, or sexual harassment, as part of this definition.

### 3.5. Violence Incidents

**Figure 1:**
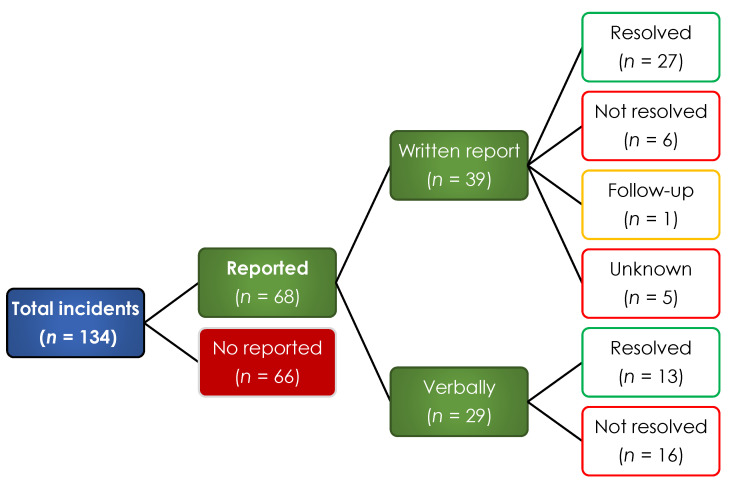
Distribution of incidents of violence reported by SSMIs in the state of Zacatecas (*n* = 157).

**Figure 2:**
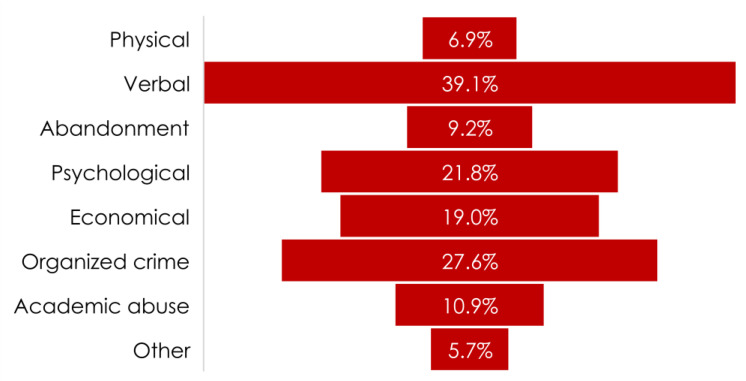
Frequency of the main types of violence identified by SSMIs (*n* = 157). As a single participant could be a victim of more than one type of violence, each bar represents an independent frequency calculated based on the total number of incidents.

A total of 119 (75.8%) of the SSMI who participated in the study stated that they were victims of some type of aggression and described a total of 134 incidents of violence, only 68 of these incidents were officially reported (Figure 1 and Figure 2). The top 5 types of violence experienced by SSMIs were verbal violence (39.1%), violence from organized crime (27.6%), psychological violence (21.8%), economic (19%) and academic abuse (10.9%) (see Figure 2). At the time of the assault, only 53 (33.6%) of the SSMIs issued an official report to the authorities, resulting in a total of 68 reports and only 39 (59.1%) of them were written reports (Figure 1).

Table 1 shows the frequency of violence incidents officially reported to an authority by SSMIs, distributed by gender. Considering the 68 issued reports, 31.9% were related to organized crime. The cases of sexual harassment represented 11.1% of the incidents and in 100% of the cases of sexual harassment, the victims were women. Thus, being a woman increased the risk of suffering sexual harassment in the studied population (*p* = 0.039). 87.5% of harassment cases came from co-workers and 12.5% came from organized crime. 75.5% of the violent incidents occurred within working hours.

**Figure 3:**
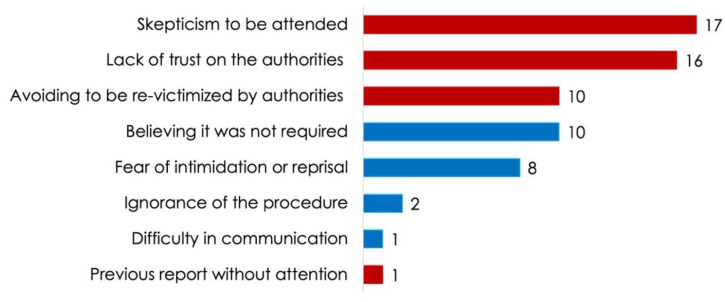
Reasons why social service interns, despite the fact of being victims of violence, do not issue formal reports (*n* = 66). Causes attributable to authority are grouped in red and unrelated ones in blue.

Figure 3 shows the most frequent reasons why SSMIs, despite the fact of being victims of some type of violence, decided not to report the incident to the authorities.

### 3.6. Violence Incidents Follow-Up

Considering the 68 violent incidents reported to the authorities, 40 of them were attended and resolved, 19 were not attended, 1 incident was still under investigation at the time of the study and for 5 incidents the SSMIs did not know the status (Figure 1). Regarding the 39 written violence reports issued, 23 reports (58.97%) were sent to the heads/managers of the areas to the institution to which each SSMI was affiliated, 10 reports (25.6%) were sent to the University to which each SSMI was affiliated, and two reports (5.12%) were sent to the municipal police. Of all reported incidents, both the Autonomous University of Zacatecas and the Autonomous University of Durango Campus Zacatecas attended 80% of the reports received, the Zacatecas Ministry of Health attended 70% of the reports received and heads/managers of the medical units attended 65.2% of the reports received.

### 3.7. Perception of Safety and Insecurity a SSMI in Its Medical Unit and Municipality

Regarding general perception security of the social service practice, 25.5% of the participants considered it very safe, 47.1% considered it somewhat secure, 15.9% considered it unsafe and 8.9% considered it as not at all secure. Supplementary Figure S1 shows a guide map and a colorimetric view of these results distributed by the municipality in which the participants carried out their social service. 

**Figure 4:**
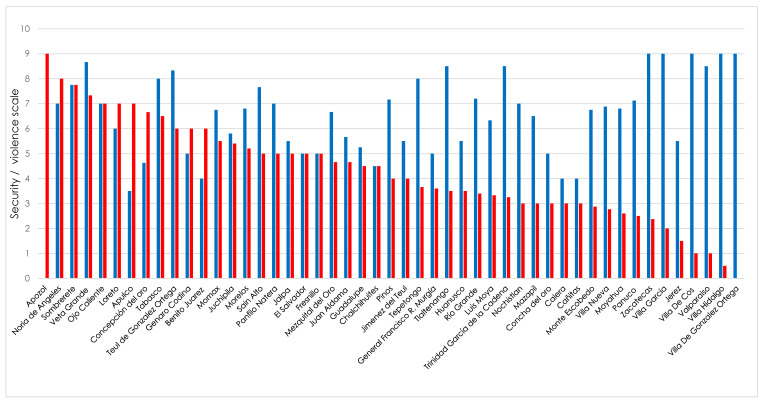
Levels of security and violence perceived by SSMIs in the state of Zacatecas in the 2020–2021 class on the medical units where they did their social service work. Plot was constructed without distinguishing by sex the study population. Security level: blue bars. Violence level: red bars.

**Figure 5:**
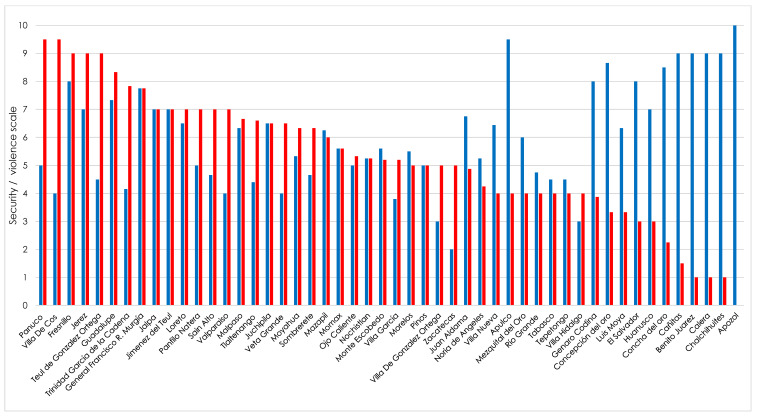
Levels of security and violence perceived by SSMIs of the State of Zacatecas of the 2020–2021 Class in the locality where they did their social service work. Plot was constructed without distinguishing by sex the study population. Security level: blue bars. Violence level: red bars.

We asked SSMIs to rate on a scale from 1 to 10, the level of perceived security in medical units and the localities where they are located. The obtained rates were grouped by municipality to get a set of average values. Figure 4 and Figure 5 show the average values obtained for medical units and municipalities, respectively. 

**Figure 6:**
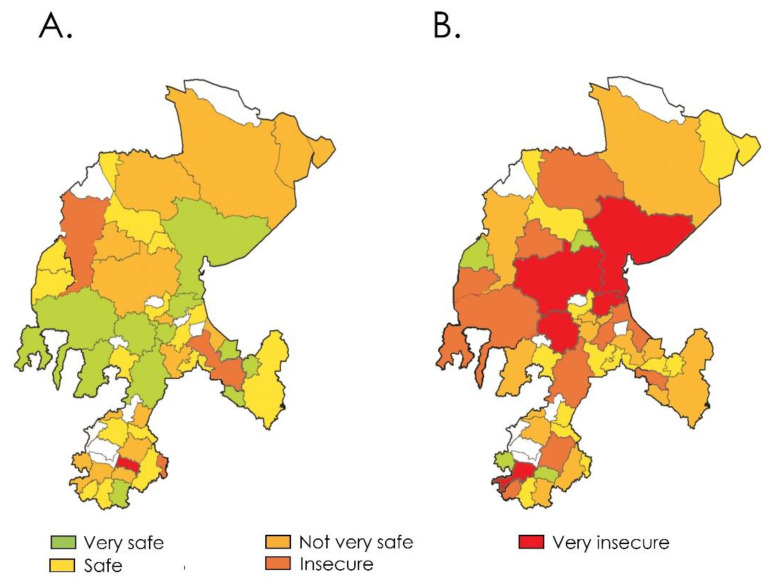
Level of security perceived by SSMIs in medical units and municipalities of the state of Zacatecas. The Figure shows a colorimetric representation of the municipalities according to the perception of safety that the SSMIs had on the medical units (**A**) and on the municipalities where these units were located (**B**). See the guide map (Supplementary Figure S1) for the identification of the municipalities.

To obtain a colorimetric representation of the obtained perception scales, the averages of the perception scales were ranged as follows: ranges 1–2, 3–4, 5–6, 7–8 and 9–10 were categorized as Very Safe, Safe, Unsafe, and Very Unsafe, respectively. Figure 6 shows the results represented on color maps. See the guide map (Supplementary Figure S1) for the identification of the municipalities. Figure 6A displays the medical units according to the perception of safety that the SSMIs had. A total of 12 (7.6%) SSMIs categorized the medical units as Very safe, 31 (19.7%) as Safe, 24 (15.3%) as Unsafe and 56 (35.7%) as Very unsafe. As shown in Figure 6B, at the localities level, a total of 26 (16.6%) SSMIs classified the those where they performed their social service as Very safe, 40 (25.5%) as Safe, 28 (17.8%) as Not very safe, 33 (21%) as Insecure and 30 (19.1%) as Very insecure.

### 3.8. SSMIs Recommendations for Improving Social Service Practice

**Figure 7:**
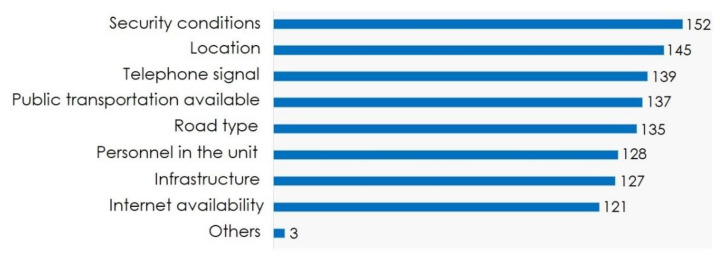
Information that SSMIS from the state of Zacatecas consider necessary to know before choosing a social service position (*n* = 157), and the frequency of responses obtained by category.

The survey applied to SSMIs included a section where they could write observations and suggestions for improving social service management processes, allocation of positions and formal procedures. All SSMIs considered it important to communicate relevant related information before the allocation of positions. The relevant information required included, medical units’ security conditions (96.8%), medical units’ location (92.4%), access to telephony network (88.5%), availability of public transportation (87.3%), type of transit roads (85.98%), type of personnel at the medical unit (81.5%), among others (Figure 7).

SSMIs considered that there are aspects in medical units that should be qualified at the end of the social service period by them, for example, medical units’ location (71.3%), transportation (means, security, and time length) (63.1%), availability of communication means (61.8%), medical units’ infrastructure (56.7%), personnel at the medical unit they worked with (52.2%), as well as public services at the municipality and/or localities (47.8%). Regarding the tools they consider necessary to improve their social service practice, 59.9% of the SSMIs expressed the need for information before choosing medical units, 59.2% of the SSMIs asked for economic transportation support, 30.6% of the SSMIs expressed the need to improve security at medical units, 8.51% suggested to cancel night shifts, 7% of the SSMIs suggested the elaboration and execution security protocols to follow when feeling in danger, 7% asked to improve conditions of medical units, 5.1% suggested not to consider medical unit in insecure locations in the process and 4.45% emphasized to improve scholarships. Only 3.2% of the participants expressed being satisfied with the conditions of social service practice in the medical unit and the conditions of the municipality and/or locality.

## 4. Discussion

Tackling abuse perception is a challenge for educational institutions as it differs at different stages of medical training and the type of violence to which SSMIs are exposed may change after they are in a professional clinical environment [14,15]. The increase of insecurity levels in Mexico as well as the fact that violence is a frequent experience among health personnel motivated this study in which the focus is given to measure the perception of security and violence at the medical units, municipalities and localities where SSMIs of Zacatecas state do social service work and to identify the main factors that lead to such perception. In the study, the most frequently officially reported acts of violence by the SSMIs were those related to organized crime (32%), verbal violence (20.6%), violence by the authorities (14.7%) and sexual harassment (11.8%). Although violent episodes against SSMIs performed by organized crime may reflect the increase of violence that exists in Zacatecas state, which we discuss next in this section, verbal violence and those episodes in which authorities are involved does not deserve less attention.

Evidence shows that extreme workload in medical training is related to SSMIs emotional exhaustion, empathy decrease, rude behavior and lack of professionalism when dealing with patients [24], all the former contributes to increasing the risk of SSMIs becoming perpetrators or victims of abuse. Besides, the lack of personal and team equipment, training to handle violent episodes, as well as the lack of programs to prevent and eliminate violence and the lack of evaluation and monitoring of this type of events, are factors that promote the occurrence and repetition of violence against SSMIs in their working environments [25]. Our results exhibit sexual harassment as one of the most frequent violent episodes and in all the reported cases, the victims were women. Thus, we conclude that being a woman is considered a risk factor for sexual harassment. These results match those reported by previous research, which alludes to the high rates of sexual harassment in medical students. It is considered that the dependence of medical students on their supervisors, particularly women medical students, is a risk factor for sexual harassment and gender discrimination, which promotes exposure to abuse during medical training [26,27,28,29].

Violent episodes against doctors in their workspaces are not only resulting from being close to places where violent interactions occur. There are also indirect factors, such as poor infrastructure conditions of medical units, lack of personal and team protection equipment, poor access to communication means, and/or the absence of formal security personnel or systems, that may increase the risk of becoming a victim of violence. In our study, it was detected that only 30% of the medical units had boundary fences or walls, only 40% of them had protected windows, only 39% of the units had an on-site telephone line, 20% of the medical units did not have access to the mobile telephony network, 26.8% of the medical units had radio equipment as the unique communication mean and, a very shocking finding was that in 5% of the medical units have no means of communication, which goes against to what is stated by official health regulations [2,23,30]. Today, and considering that SSMIs are a very important stage of his/her formation, access to the Internet and telephony networks is very important not only because they are means to information access but also because they are needed to communicate problems, patient transfers and other relevant events. Although access to the Internet and telephony networks is in several rural communities in Mexico, there are cases where these accesses are available to the medical units in these communities via specific government programs [31,32]. Thus, all the above-described shows that the improvement of infrastructure and the access to communication means in medical units constitute area aspects to be reinforced to contribute to preventing SSMIs’ exposition to acts of violence.

During their one-year period of public service, SSMIs often need to travel to the assigned medical units. In Mexico in the last years, the number of reports of cases of assaults and murders of doctors in the localities or on the way to the communities where they performed their social service [25] has increased, which often results in SSMIs leaving rural locations because of the fear of becoming victims of organized crime [9]. In our study, more than 40% of the interviewees rated the journey to medical units from not at all safe, to not very safe, due to the presence of organized crime and/or violence episodes caused by them. Besides, the obtained data on safety/insecurity perception of medical units locations shows that those located in the municipalities of Apozol, Noria de Ángeles and Sombrerete were considered as the most insecure and the municipalities of Pánuco, Villa de Cos and Fresnillo as the ones with the highest levels of insecurity. Our results are similar to those reported by INEGI, where 94.3% of surveyed people (aged 18 years and over) considered that living in Fresnillo is unsafe [33]. Previous studies have reported that 30% of graduates doing social service know in advance the assigned locality [2,34], however, none of them know the real magnitude of the levels of violence and crime at the locality, until they begin their social practice [2,34]. Thus, it is very important to change the status quo and generate tools and mechanisms to allow SSMIs to have a priori the required information to better choose the medical unit.

In Mexico, it is an uncommon practice to report offenses (independently of whether they involved violence or not). It is known that of all Mexicans who are victims of violence, 92.4% do not report the incidents for reasons attributable to the authority (64.1%) [35]. Our results are similar to these statistics as 75.8% of the SSMIs who participated in the study stated that they have suffered from violence of some sort during their social service practice, but only 33.6% of them reported the incident. The lack of interest in issuing a report is related to causes attributable to the authority in 67.7% of the cases, particularly skepticism to being attended, lack of trust and/or not wanting to be re-victimized. The absence of formal crime reports as well as the increase of violence episodes experienced by SSMIs, exhibit an urgent need to strengthen actions for reducing the acts of violence in their work environments, improving follow-up processes until their resolution (since 47% of the incidents were not resolved). Besides, these actions should aim to improve the perception of the prosecution and administration of justice to reduce trust gaps among SSMIs and authorities.

Violence is not an intrinsic part of the human condition, because as with many threats to public health, its impact can be prevented and reduced [36]. Thus, it is a challenge for university and health authorities in Mexico to properly classify the risks of violence of various kinds that occur in the medical units where SSMIs work. Besides, to promote a culture of violence prevention, and improve the infrastructure and communication means conditions of medical units in rural areas to preserve the physical and mental integrity of the SSMIs. Similarly, it is a priority to generate strategies and plans to prevent and/or reduce the risk of exposure of SSMIs to violent episodes in the medical units and the communities where they are located. 

### Limitations 

It is important to highlight that one of the main limitations of the study is related to the “perception” concept *per se*, because the perception of an individual is subjective, selective, and temporal. Therefore, the perception of insecurity reported in this study could be biased by the relationships that SSMIs had with administrators, managers and other personnel in medical units, the exposure of SSMIs to news and/or advertisements on criminal acts happening in Mexico and the state of Zacatecas, the events that they have experienced during their social service practice and the way in which these events impacted their mental health, etc. Thus, considering that the number of participants for each medical unit is small it could be important to periodically carry out follow-up studies to assess the main variables that influence SSMIs’ perception of insecurity and violence.

## 5. Conclusions

A high proportion of the SSMIs included in the study (75.8%) were victims of violence during their social service practice, by organized crime, by the authorities and/or co-workers. Being a woman increased the risk of sexual harassment among the population studied. A high percentage of SSMIs (66.4%) do not report acts of violence, mainly for causes related to the trust in authorities. The poor physical infrastructure and the lack of communication means in the medical units are aspects to solve that require special attention in the state of Zacatecas. Thus, it becomes a priority to generate and coordinate strategies to prevent and reduce the risk of violence exposure in medical units and the places where they are located, as well as to efficiently process formal violence reports to promote a safe environment that favors the fulfillment of the practice of SSMIs in Mexico. By measuring the perception of security and violence in this study, we want to provide a preliminary diagnose that drives the generation of action plans to improve aspects related to such a perception in the short, medium, and long term.

## Figures and Tables

**Table 1:** Official report on the frequency of violence incidents of SSMIs, classified by sex. A total of 53 SSMIs (18 men, 33 women, and 2 who did not specify sex) reported a total of 68 incidents.

Type of Reported Incident	Number of Reported Incidents	Reported Incidents		
		Women	Men	Not Specified ^2^
Assaults from Organized ^1^ Crime	23 (33.8)	12 (17.6)	10 (14.7)	1 (1.5)
Verbal violence	14 (20.6)	6 (8.8)	5 (7.4)	3 (4.4)
Violence by the Authorities	10 (14.7)	5 (7.4)	2 (2.9)	0
Sexual harassment	8 (11.8)	8 (11.8)	0	0
Forced entry	5 (7.4)	3 (4.4)	2 (2.9)	0
Robbery	4 (5.9)	2 (2.9)	2 (2.9)	0
Harassment out of working hours	3 (4.41)	1 (1.5)	2 (2.9)	0
Property damage	1 (1.47)	1 (1.5)	0	0
Total	68 (100)	38 (55.9)	26 (38.2)	4 (5.9)

^1^ All incidents that included threats with firearms, kidnapping, extortion, use of physical force, encounters of criminal groups, criminal groups checkpoints and criminal groups posing as official authorities are included. ^2^ Two participants did not specify their gender.

## Data Availability

All data supporting reported results are included in the manuscript. Additional information regarding data that support the findings of this study will be available from the corresponding author [M.L.M.-F.] and [I.G.-V.], upon reasonable request.

## Supplementary Materials

The following are available online at https://www.mdpi.com/article/10.3390/ijerph19010318/s1, Figure S1: General safety perception of the medical social service in the state of Zacatecas.
