# Supplementary material for: Security and Violence Perception of Medical Interns during Social Service Practice in Mexico
Source: Int J Environ Res Public Health. 2021 Dec 29;19(1):318. doi: 10.3390/ijerph19010318 (PMC8751139; doi:10.3390/ijerph19010318)
Supplement: Supplementary file 1 [file ijerph-19-00318-s001.zip › ijerph-1488253-supplementary.pdf]

Supplementary material

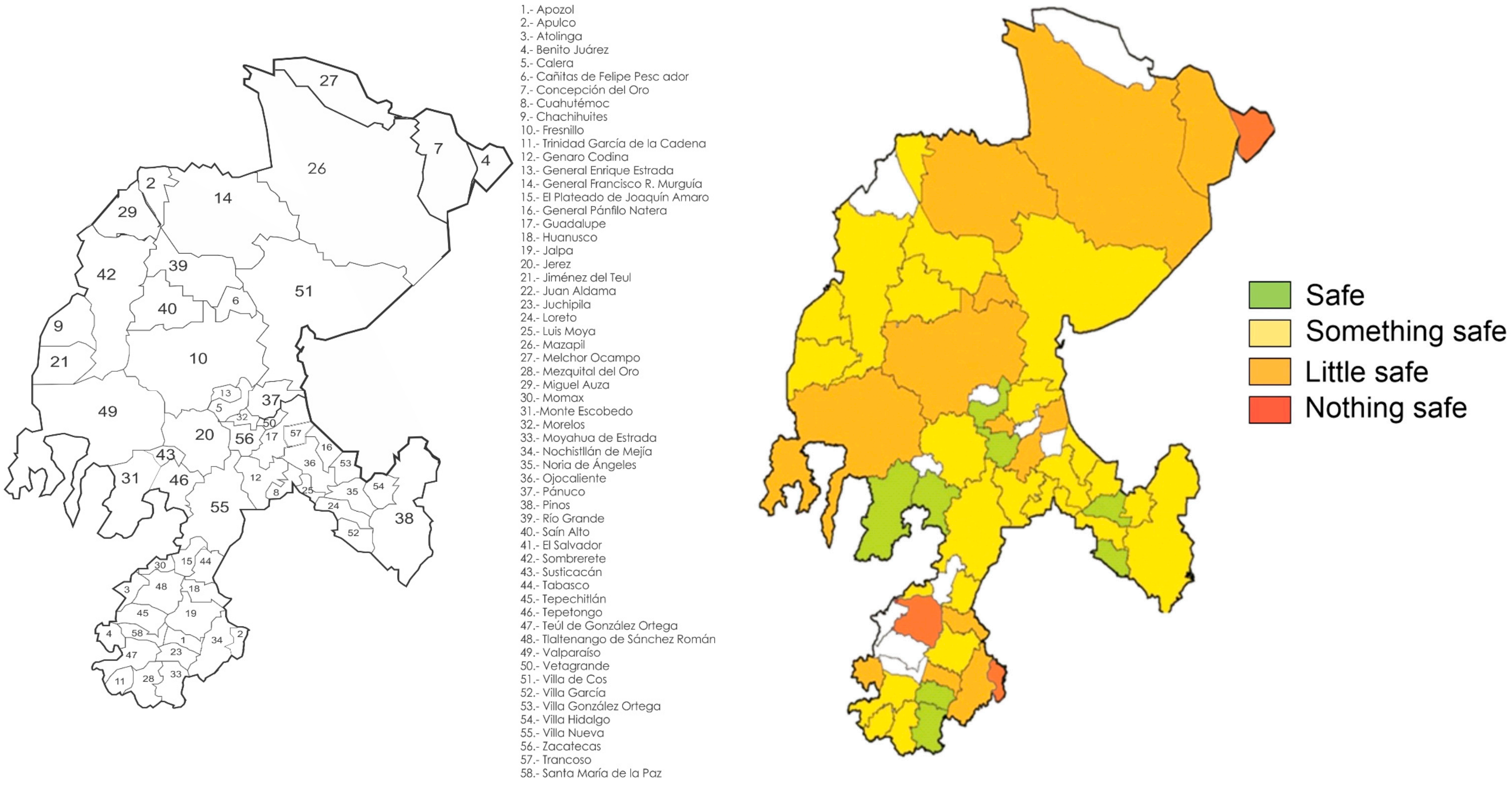

A. **B.**

Figure S1. General safety perception of the medical social service in the state of Zacatecas. (A). Guide map and (B) Colorimetric representation of the municipalities of the state of Zacatecas according to the general safety perception that medical interns of social service in the state of Zacatecas felt during the performance of their social work.
